# Optimizing Photobiomodulation Radiometric and Spectral Parameters In Vitro to Enhance Angiogenesis and Mitochondrial Function

**DOI:** 10.3390/ijms26010093

**Published:** 2024-12-26

**Authors:** Jaroslava Joniová, Aurélien Gregor, Martine Lambelet, Sébastien Déglise, Florent Allagnat, Georges Wagnières

**Affiliations:** 1 Laboratory for Functional and Metabolic Imaging (LIFMET), Institute of Physics, Swiss Federal Institute of Technology (EPFL), Station 3, 1015 Lausanne, Switzerlandgeorges.wagnieres@epfl.ch (G.W.); 2 Department of Vascular Surgery, Lausanne University Hospital (CHUV), 1005 Lausanne, Switzerland; martine.lambelet@chuv.ch (M.L.); florent.allagnat@chuv.ch (F.A.)

**Keywords:** photobiomodulation, PBM, angiogenesis, cell migration, endothelial cells, protoporphyrin IX, mitochondrial metabolic activity

## Abstract

Photobiomodulation (PBM) therapy, a therapeutic approach utilizing low-level light, has garnered significant attention for its potential to modulate various biological processes. This study aimed at optimizing and investigating the effects of PBM on angiogenesis and mitochondrial metabolic activity. In vitro experiments using human umbilical vein endothelial cells (HUVECs) and vascular smooth muscle cells (VSMCs) were performed to assess PBM’s impacts on cell migration, proliferation, endogenous protoporphyrin IX production, mitochondrial membrane potential, Rhodamine 123 fluorescence lifetime, mitochondrial morphology, and oxygen consumption. Our findings demonstrated that the PBM approach significantly stimulates HUVECs and VSMCs, highlighting the importance of precise light dosimetry for optimal outcomes. Interestingly, our results indicate that in our conditions, the optimal radiometric and spectral parameters are similar for HUVECs and VSMCs for the different endpoints mentioned above. In conclusion, our study strongly suggests that PBM holds promise as a therapeutic intervention for conditions characterized by impaired angiogenesis, such as wound healing, ischemia, and cardiovascular disease. Further research is necessary to fully elucidate the underlying mechanisms and optimize the radiometric and spectral parameters for clinical applications.

## 1. Introduction

Angiogenesis, the formation of new blood vessels from pre-existing ones, is a crucial process involved in the proliferative phase of wound healing. It provides the wound site with oxygen and essential nutrients necessary for tissue repair and cell proliferation. This vascularization process promotes cellular migration and matrix formation enables the transport of immune cells, which is crucial for defense against infection. Insufficient angiogenesis has been linked to chronic wound conditions, such as diabetic ulcers, and ischemic diseases, including coronary artery disease, peripheral arterial disease, stroke, and acute limb ischemia, where inadequate blood supply hinders the healing process. In vitro, studies have been instrumental in elucidating the mechanisms underlying angiogenesis and its role in wound healing [1,2,3]. By utilizing controlled experimental conditions and advanced in vivo techniques, the various aspects of angiogenesis and mitochondrial metabolism can be assessed.

Photobiomodulation (PBM) therapy is a non-invasive treatment using light in the red or near-infrared parts of the spectrum. With over five decades of research, PBM has demonstrated broad therapeutic potential, including wound healing. Numerous studies have confirmed PBM’s ability to stimulate cellular processes essential for wound repair, both in laboratory and living organisms [4]. It was observed that PBM can increase adenosine triphosphate (ATP) production, activate various secondary messengers, and upregulate cascades of intracellular signals [4,5,6]. Another theory is that cytochrome c oxidase (CcO) is the main acceptor of photons responsible for the facilitation of electron transfer to molecular oxygen and, hence, an increase in the mitochondrial respiratory chain that ultimately leads to increased mitochondrial activity, including the ATP production and accelerated oxidative metabolism [4,7,8,9]. Other examined mechanisms include the increase and/or decrease in the reactive oxygen species (ROS) production, intracellular calcium concentrations, and nitric oxide (NO) production after PBM [4,10,11]. Clinical studies have shown that PBM promotes angiogenesis, reduces inflammation, and accelerates the wound healing phases, which is critical for treating chronic and difficult-to-heal wounds such as diabetic ulcers and can significantly improve healing outcomes and reduce recovery time [12,13].

Despite extensive research, the precise mechanisms underlying PBM remain unclear, and the optimal radiometric parameters for enhancing PBM-induced angiogenesis still need to be fully defined. Although PBM therapy holds promise in wound healing, several challenges persist. A significant issue is the lack of standardized protocols regarding parameters like wavelength, energy dose, and treatment duration, which are crucial for consistent outcomes [14]. To address these limitations, our study implemented meticulous controls and optimized radiometric and spectral conditions. We aimed to optimize and investigate the effects of PBM in vitro on cell migration, proliferation, and mitochondrial function, both of which are essential for tissue repair and regeneration. By optimizing radiometric parameters such as wavelength and irradiance, we sought to identify the most effective conditions for stimulating the proliferation and migration of primary human umbilical vein endothelial cells (HUVECs) and vascular smooth muscle cells (VSMCs).

HUVECs are the primary drivers of angiogenesis, forming the inner lining of blood vessels. They respond to pro-angiogenic signals by proliferating, migrating, and forming tubular structures. They initiate the sprouting of new blood vessels and are essential for establishing the vascular network [15]. Conversely, VSMCs are primarily involved in arteriogenesis (the formation of larger vessels) rather than capillary-level angiogenesis. VSMCs provide structural integrity and contractile ability to larger blood vessels, helping regulate vascular tone and blood pressure. While they do not directly contribute to capillary sprouting, they are critical for the stability and function of larger vessels and are involved in the maturation and remodeling stages of vessel formation after the initial angiogenic events have occurred [16]. Endogenous PpIX production, mitochondrial membrane potential, Rhod 123 fluorescence lifetime, alterations of the mitochondrial morphology, and oxygen consumption rate (OCR) were also assessed. Our results highlight the significant potential of PBM to stimulate endothelial and smooth muscle cell function. Moreover, our study emphasizes the critical role of precise light dosimetry for optimal therapeutic outcomes. Although further research is necessary to elucidate the underlying mechanisms and optimize PBM parameters for clinical application, our results highlight the potential of PBM as a treatment for conditions characterized by impaired angiogenesis.

## 2. Results

### 2.1. PBM Enhances Cell Migration and Proliferation in HUVECs and VSMCs

**Figure 1:**
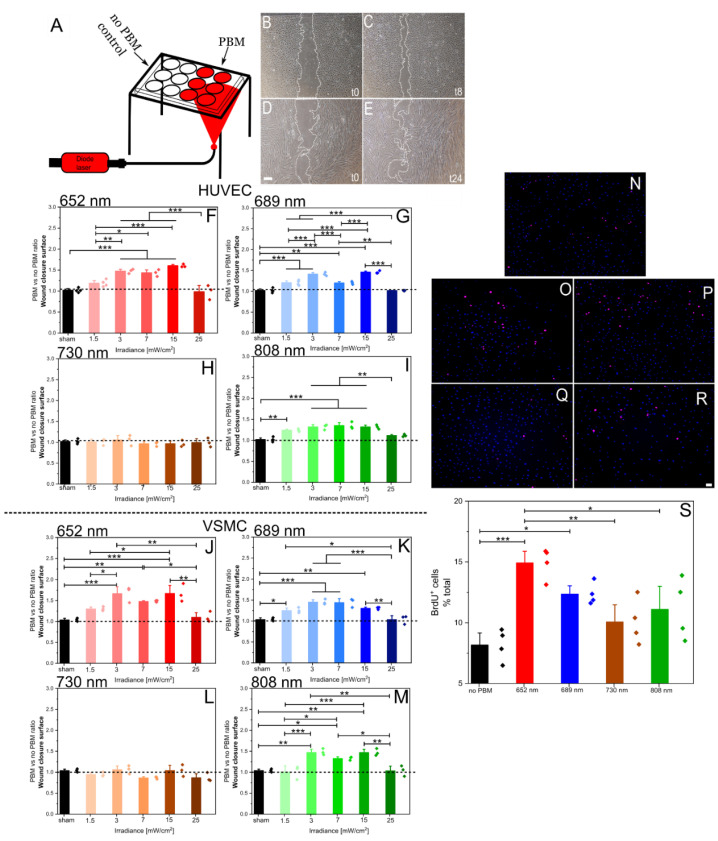
PBM illumination setup and effects on cell migration and proliferation. (**A**): Schematic representation of the PBM illumination setup. (**B**–**E**): Representative images of wound healing assay, 15 mW/cm^2^ for 180 s at 652 nm in the HUVECs (**B**,**C**) and VSMCs (**D**,**E**) after 8 and 24 h, respectively. Scale bar: 100 µm. (**F**–**M**): PBM effects on cell migration in the HUVECs (**F**–**I**) and VSMCs (**J**–**M**) expressed as the PBM/no-PBM ratio of wound closure surface. The dotted line indicates the value of this ratio if no PBM (sham) would be applied. Data represent mean ± SEM (n ≥ 3 independent measurements). * *p* < 0.05, ** *p* < 0.01, and *** *p* < 0.001, as determined by one-way ANOVA with post hoc *t*-tests with Tukey’s correction for multiple comparisons. (**N**–**R**): Representative images of BrdU-positive HUVEC (pink) and DAPI-positive nuclei (blue) after different PBM conditions. (**N**): no PBM, 15 mW/cm^2^ for 180 s at (**O**): 652 nm, (**P**): 689 nm, (**Q**): 730 nm, and (**R**): 808 nm. The scale bar represents 50 µm. (**S**): PBM effects on HUVEC proliferation assessed by BrdU incorporation (BrdU-positive cells/DAPI-positive nuclei). Data represent mean ± SEM (n = 4). * *p* < 0.05, ** *p* < 0.01, and *** *p* < 0.001, as determined by one-way ANOVA with post hoc *t*-tests with Tukey’s correction for multiple comparisons.

To investigate the influence of PBM on cell migration, HUVECs and VSMCs were subjected to a wound healing assay. A range of wavelengths (652, 689, 730, and 808 nm) and irradiances (1.5; 3; 7; 15; and 25 mW/cm^2^) were tested, maintaining a constant illumination duration of 180 s based on our previous studies [17,18]. Figure 1A illustrates the experimental setup. Representative images showcasing the wound healing process for the HUVECs (Figure 1B,C) and VSMCs (Figure 1D,E) are presented. The PBM effect on the cell migration was found to vary according to both the wavelength and irradiance applied.

For the HUVECs, PBM conditions at 652, 689, and 808 nm (Figure 1F,G,I) with irradiances between 3 and 15 mW/cm^2^ produced the most significant enhancement in cell migration, demonstrating a strong dose-dependent effect within this range. At lower irradiances (1.5 mW/cm^2^), the PBM effect on migration was reduced, while at higher irradiances (25 mW/cm^2^), the stimulatory effect diminished. Similar results were observed for the VSMCs (Figure 1J,K,M). The most pronounced enhancement for both cell types occurred at 652 nm with an irradiance of 15 mW/cm^2^, as illustrated in Figure 1F,J. The dotted lines indicate the PBM effect if no PBM (sham) is applied. In addition, illumination at 730 nm did not significantly influence cell migration across any of the tested irradiance levels, as shown in Figure 1H,L.

Furthermore, to determine whether the effects of PBM extend beyond cell migration and stimulate cell proliferation, a BrdU incorporation assay was conducted on the HUVECs (Figure 1N–R). As shown in Figure 1S, significant changes in HUVEC proliferation were observed under the PBM conditions of 15 mW/cm^2^ for 180 s at wavelengths of 652 nm and 689 nm, with a less pronounced effect at 808 nm. Notably, illumination at 730 nm did not affect cell proliferation.

PBM modulates mitochondrial metabolism as evidenced by PpIX endogenous production and mitochondrial potential.

**Figure 2:**
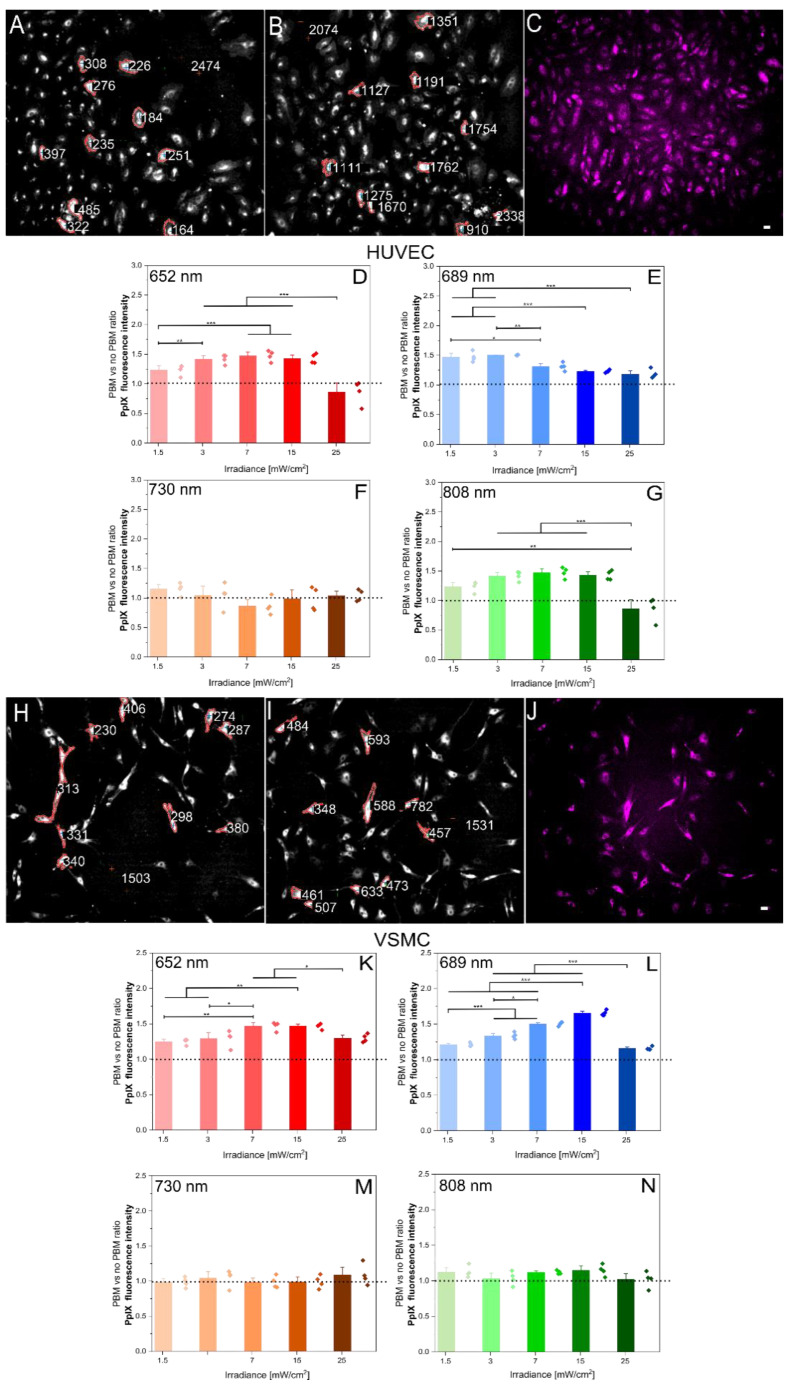
PBM effect on the endogenous PpIX in the HUVECs and VSMCs. (**A**,**B**): Representative PpIX image processing analysis of the HUVECs for no PBM (**A**) and PBM (**B**)-treated cells. The values represent the mean pixel intensities within the region of interest (outlined by the red contour). (**C**): Representative fluorescent image of endogenous PpIX production in the HUVECs after 3 h incubation with 1 mM ALA. The scale bar represents 50 µm. (**D**–**G**): PBM-induced changes in the HUVEC PpIX production (PBM/no PBM ratio) at different wavelengths. The dotted line indicates the value of this ratio if no PBM (sham) would be applied. Data represent mean ± SEM (n ≥ 60 cells). (**H**–**N**): Corresponding representative images and analyses for the VSMCs. * *p* < 0.05, ** *p* < 0.01, and *** *p* < 0.001, as determined by one-way ANOVA with post hoc *t*-tests with Tukey’s correction for multiple comparisons.

Two distinct methods were employed to investigate the influence of PBM on mitochondrial activity. First, the endogenous PpIX production was assessed in both the HUVECs and VSMCs using the same PBM parameters as in the cell migration study. Figure 2A–G presents representative image processing and fluorescence data for PpIX in the HUVECs, while Figure 2H–N depicts the corresponding data for the VSMCs.

**Figure 3:**
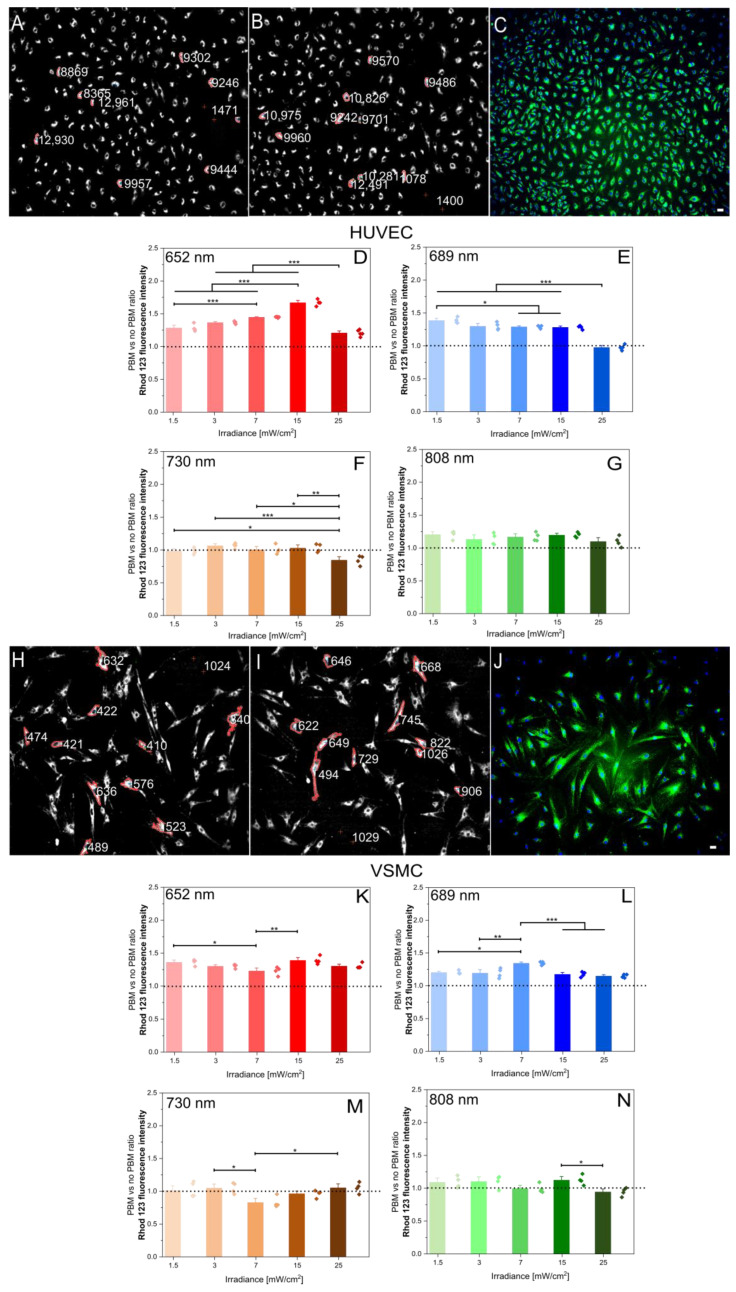
PBM effect on the mitochondrial potential in the HUVECs and VSMCs. (**A**,**B**): Representative Rhod 123 image processing analysis in the HUVECs for no PBM (**A**) and PBM (**B**). The values represent the mean pixel intensities within the region of interest (outlined by the red contour). (**C**): Representative fluorescent image of Rhod 123 in the HUVECs after 30 min incubation with 2.6 µM Rhod 123. The scale bar represents 50 µm. (**D**–**G**): PBM-induced changes in the HUVEC mitochondrial potential (PBM/no PBM ratio) at different wavelengths. The dotted line indicates the value of this ratio if no PBM (sham) would be applied. Data represent mean ± SEM (n ≥ 60 cells). (**H**–**N**): Corresponding representative images and analyses for the VSMCs. * *p* < 0.05, ** *p* < 0.01, and *** *p* < 0.001, as determined by one-way ANOVA with post hoc *t*-tests with Tukey’s correction for multiple comparisons.

Secondly, mitochondrial potential was monitored by Rhod 123 fluorescence quantification in both cell lines using the same parameters as above. Similarly, Figure 3A–G shows representative image processing and fluorescence data for Rhod 123 in the HUVECs, while Figure 3H–N represents the corresponding data for the VSMCs.

As shown in Figure 2D–G,K–N and Figure 3D–G,K–N, significant PBM effects were observed at various wavelengths within the range of 1.5 to 15 mW/cm^2^. The most potent effect was achieved at 652 nm with an irradiance of 15 mW/cm^2^ for 180 s. This led to increased mitochondrial metabolism in both cell lines, as evidenced by both PpIX production and mitochondrial membrane potential. In contrast, the 730 nm wavelength did not elicit significant changes in either parameter under any tested condition. Surprisingly, 808 nm had only a minimal effect on endogenous PpIX production in the VSMCs and mitochondrial membrane potential in both cell lines.

To analyze fluorescent images of PpIX and Rhod 123, a semi-automated pipeline based on Bradley’s adaptive thresholding was developed [19,20]. This method effectively segments and quantifies the fluorescence intensity in various cell lines, including human glioblastoma (U87-MG), HCM, HUVEC, and VSMC. To validate the semi-automated method, a manual segmentation approach was employed [17,18]. A quantitative fluorescence analysis using ImageJ was performed on the same cells using both methods. The average difference in mean fluorescence ratio (PBM/no-PBM) between the two methods was 6% [18], indicating a strong correlation between the semi-automated and manual methods. This semi-automated method offers a reliable and efficient approach for analyzing fluorescence images, providing accurate and consistent results. Its applicability to various cell lines demonstrates its versatility and potential for broader use in biomedical research.

### 2.2. PBM Alters the Rhod 123 Fluorescence Lifetime in the Mitochondria

**Figure 4:**
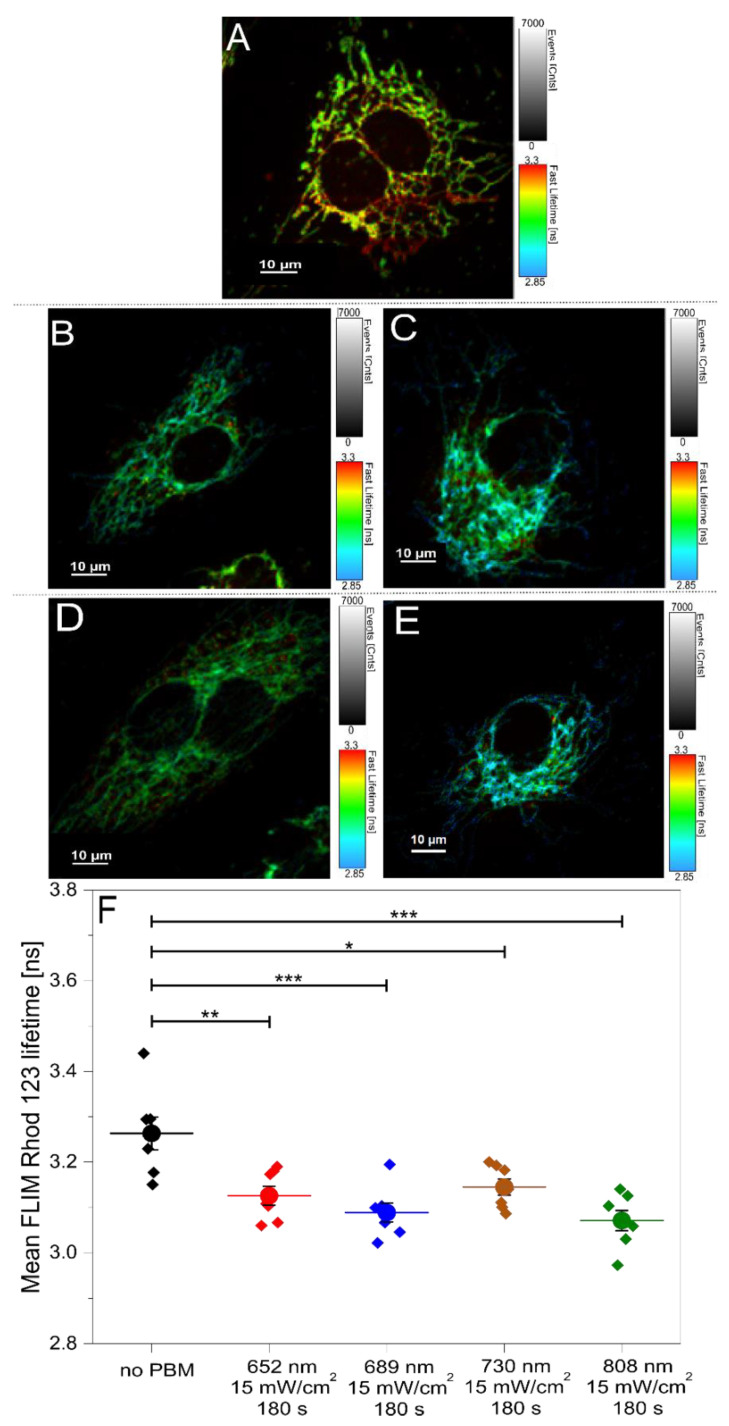
Representative FLIM images and mean Rhod-123 lifetimes following PBM illumination. (**A**): no PBM; 15 mW/cm^2^ for 180 s at (**B**): 652 nm, (**C**): 689 nm, (**D**): 730 nm, and (**E**): 808 nm; and (**F**): average Rhod 123 lifetimes and respective SEMs determined by the analysis of the FLIM images after selected PBM treatments. Data represent mean ± SEM (n ≥ 6 images corresponding to18 ROIs). * *p* < 0.05, ** *p* < 0.01, and *** *p* < 0.001, as determined by one-way ANOVA with post hoc *t*-tests with Tukey’s correction for multiple comparisons.

In subsequent experiments, our focus shifted to studying the PBM effect, specifically in the HUVECs, due to their predominant role in angiogenesis [21,22,23]. Rhod 123 FLIM was performed to assess PBM’s influence on mitochondrial function. Given the previous determination of 15 mW/cm^2^ for 180 s as the optimal PBM condition, this parameter was applied for all the wavelengths in this study. Figure 4A–E presents the representative FLIM images demonstrating the impact of different PBM conditions on Rhod 123 fluorescence lifetime. Figure 4F summarizes the average lifetimes calculated from these images.

In our conditions, the average Rhod 123 fluorescence lifetime in the HUVECs was determined to be 3.263 ± 0.036 ns under the control condition (no PBM). Exposure to PBM at all the tested wavelengths resulted in a significant decrease in Rhod 123 lifetime compared to the control. Specifically, lifetimes were 3.126 ± 0.021 ns (652 nm), 3.088 ± 0.021 ns (689 nm), and 3.071 ± 0.022 ns (808 nm). Unexpectedly, even at 730 nm, a significant shift in the Rhod 123 lifetime was observed, measuring 3.145 ± 0.018 ns.

### 2.3. PBM Changes the Mitochondrial Morphology and Network Connectivity in HUVECs

**Figure 5:**
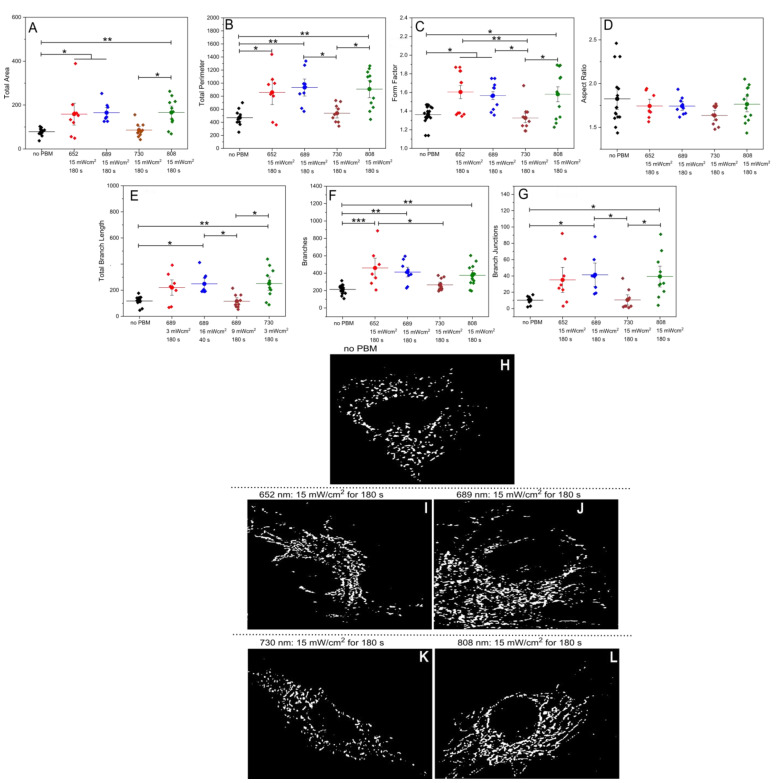
PBM effects on the mitochondrial morphology and network connectivity. Two-dimensionalmorphological analysis comparing mitochondrial network connectivity after different PBM illuminations within each morphological category: (**A**): total area, (**B**): total perimeter, (**C**): form factor, (**D**): aspect ratio, (**E**): total branch length, (**F**): branches, and (**G**): branch junctions. At least 8 images per condition were analyzed. * *p* < 0.05, ** *p* < 0.01, and *** *p* < 0.001, as determined by one-way ANOVA with post hoc *t*-tests with Tukey’s correction for multiple comparisons. (**H**–**L**): Representative images of the objects identified through adaptive thresholding.

To better understand the mitochondrial dynamics in the HUVECs, we conducted a comprehensive quantitative analysis of the FLIM fluorescence intensity images of Rhod 123-stained cells. Figure 5A–G demonstrates the PBM effects on the morphology and connectivity of the mitochondrial network, which were categorized into various morphological parameters. These include the total area, perimeter, form factor, and aspect ratio, which describe mitochondrial size and shape. Additionally, we analyzed the total branch length, the number of branches, and branch junctions to evaluate the overall morphological complexity and connectivity of the mitochondrial network.

Across all the parameters used to quantify the morphology and dynamics of the mitochondrial network, we observed significant differences at all the wavelengths, except 730 nm, which did not exhibit any notable effect. Additionally, Figure 5H–L presents illustrative images obtained after applying adaptive thresholding to the images.

### 2.4. PBM Increases the OCR in HUVECs

**Figure 6:**
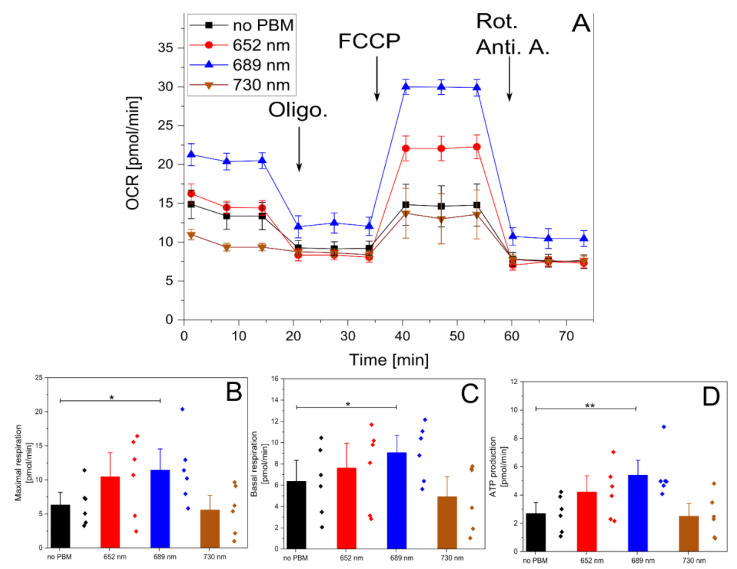
Mitochondrial stress assay monitored without or after PBM. (**A**): Representative traces of the OCR after the PBM illuminations of 15 mW/cm^2^ applied for 180 s at different wavelengths. (**B**–**D**): Quantitative assessments of OCR after the PBM illumination exposures. Data represent mean ± SEM of n = 6 independent measurements. * *p* < 0.05 and ** *p* < 0.01, as determined by one-way ANOVA with post hoc *t*-tests with Tukey’s correction for multiple comparisons.

Finally, to assess the PBM impact on cellular respiration, we performed a mitochondrial stress test in the HUVECs using a Seahorse setup (Figure 6). Building upon earlier findings, we opted to explore the impact of an irradiance of 15 mW/cm^2^ applied for 180 s at the following wavelengths: 652, 689, and 730 nm.

The 689 nm illumination significantly increased OCR, leading to higher maximal and basal respiration as well as increased ATP production. Interestingly, while 652 nm showed a tendency to increase OCR parameters, these changes were not statistically significant. 730 nm irradiation had no measurable effect on the HUVEC OCR.

The differences in OCR responses between the 652 nm and 689 nm light likely reflect distinct wavelength-specific cellular mechanisms, as OCR is not always directly linked to migration or proliferation. Both wavelengths demonstrated clear stimulatory effects on cell behavior, emphasizing their potential to influence key cellular processes through different metabolic pathways.

## 3. Discussion

Despite decades of research, the precise mechanisms underlying PBM remain to be fully elucidated. Nonetheless, PBM has emerged as a promising therapeutic technique with applications in regenerative medicine among others [24]. While PBM’s effects on inflammation, wound healing, and tissue repair have been extensively studied [4,10,25], its influence on angiogenesis continues to be a compelling area of investigation.

Angiogenesis, the formation of new blood vessels from existing ones, is a fundamental process involved in various physiological and pathological conditions [26]. This complex process is regulated by an intricate network of molecular mechanisms that are coupled with mitochondrial metabolism. In addition, certain groups reported that the cellular responses to PBM vary for different cell or tissue types and their specific functions [27,28,29]. For instance, fibroblasts, keratinocytes, and melanocytes in the skin exhibit distinct responses to red and infrared light exposure. Fibroblasts demonstrate increased metabolic activity and collagen production [30,31], while keratinocytes exhibit increased respiration and proliferation [32]. Melanocytes show more complex responses, influenced by experimental conditions and light doses [27]. In brain injury, transcranial PBM has been shown to promote cellular repair, reduce inflammation, and enhance cellular signaling mechanisms [33]. These findings highlight the tissue-specific nature of PBM effects and the importance of optimizing treatment parameters.

Our in vitro study, utilizing HUVECs and VSMCs, aimed to optimize the radiometric parameters for enhancing PBM-induced angiogenesis. The in vitro assay was established as a reliable model for extrapolating in vivo processes and studying live cell behavior [34,35]. The illumination time was fixed at 180 s, as this time was previously found to be optimal for different cell lines [19,23]. A comprehensive range of PBM conditions was explored, including four wavelengths (652, 689, 730, and 808 nm) and five irradiances (1.5, 3, 7, 17, and 25 mW/cm^2^). It should also be noted that all the PBM experiments were conducted at room temperature. To assess the potential influence of temperature, a control study was performed historically comparing illuminations inside and outside an incubator (37 °C) [18]. Comparable results were observed in both conditions [18], indicating that temperature and temperature variations had a negligible impact on our results.

In general, for the HUVECs, a bell-shaped response, often described as biphasic or bimodal [25], was observed across all the studied PBM wavelengths except 730 nm, as evidenced by the changes in cell migration, endogenous PpIX production, and mitochondrial membrane potential, indicating that the enhancement of cell migration and mitochondrial activity by PBM is optimized at specific wavelengths and irradiances. In other words, our findings further emphasize that both insufficient and excessive PBM irradiances and light doses can diminish or negate the therapeutic effects. This phenomenon aligns with well-documented previous studies [4,17,18,25]. Similar observations were made for VSMCs regarding cell migration, although the so-called biphasic response was not evident for 808 nm in the PpIX production and mitochondrial membrane potential studies. This, and other studies, suggest that PBMs performed at 808 nm often elicit different responses in HUVECs and VSMCs, particularly regarding the PpIX production [36,37]. It should be noted that under our experimental conditions, HUVECs exhibit higher metabolic activity compared to VSMCs, likely due to the fact that they are derived from younger individuals compared to VSMCs, which originate from older patients.

The lack of observable PBM effects at 730 nm aligns well with prior research [4,7,9,17,18], confirming it as an “off” wavelength. Indeed, our results are consistent with previous studies identifying CcO as the primary photoacceptor in PBM [7,38,39]. Indeed, CcO presents an absorption dip at this wavelength [7]. Interestingly, the absence of a PBM effect at 730 nm for all the irradiances (1.5 to 25 mW/cm^2^) indicates that the positive effects observed at other wavelengths are not attributable to thermal effects induced by the cell illuminations.

Our study has revealed a significant correlation between endogenous PpIX production and mitochondrial membrane potential for different PBM illuminations in both cell lines. It is well established that mitochondria play a pivotal role in the mechanisms underlying PBM [4,18]. Endogenous PpIX production is also closely linked to mitochondrial activity, as its synthesis partially occurs within the mitochondria. Previous research has demonstrated a correlation between endogenous PpIX production and other mitochondrial metabolic markers, such as mitochondrial membrane potential, oxygen consumption, and morphological changes [18]. Consequently, the simple monitoring of the PpIX production can provide valuable insights into mitochondrial metabolic activity. Furthermore, our previous works with human cardiac cells (HCM) highlighted a similar correlation between endogenous PpIX production and mitochondrial membrane potential following PBM [18]. Observing a similar phenomenon in these cell lines is intriguing and suggests a broader applicability of this relationship across different cell types. The subtle increase in PpIX production and mitochondrial membrane potential observed in the HUVECs compared to VSMCs under most PBM illumination conditions can be attributed to their differing metabolic states. As previously mentioned, the HUVECs exhibited greater metabolic activity under our experimental conditions, likely due to their younger age compared to the VSMCs.

Changes in the Rhod 123 FLIM that were observed in the HUVECs after the PBM illuminations further demonstrate the increased mitochondrial activity post-PBM since the quenching of Rhod 123 and reduced lifetimes indicate the energization and increase in mitochondrial potential, reflecting stimulation of the metabolism. FLIM, an advanced imaging technique, measures the fluorescence lifetime of molecules, providing insights into mitochondrial function and cellular metabolism. This technique enables the evaluation of molecular interactions, visualization of dynamic cellular processes, and differentiation between fluorophores [40]. Our FLIM results align with those presented in the mitochondrial membrane potential study (Figure 3) and previous findings [41]. Despite an increase in intensity, the observed decrease in Rhod 123 fluorescence lifetime in energized mitochondria is likely due to a partial quenching of the Rhod 123 fluorescence by its accumulation in mitochondria. Conversely, the Rhod 123 fluorescence lifetime is relatively independent of its concentration. The unexpected decrease in the Rhod 123 lifetime at 730 nm could be attributed to changes in the mitochondrial environment, such as alterations in viscosity, pH, or binding interactions [42]. Increased ROS production from mitochondrial dynamics may also influence the dye’s behavior without altering its accumulation [43].

Changes in mitochondrial morphology and network dynamics after PBM allowed us to quantify PBM’s effects on mitochondrial function directly. Mitochondria are crucial for cellular function, adapting to stress by producing ATP through oxidative phosphorylation [44]. Mitochondria typically exhibit a tubular shape in healthy cells and form complex networks. Conversely, stressed or dying cells often display fragmented and swollen mitochondrial networks, decreased mitochondrial membrane potential, and increased ROS production, leading to disrupted metabolism and other cellular dysfunctions [45,46,47]. Therefore, the quantitative analysis of mitochondrial morphology and network connectivity provides valuable insights into cellular physiology and pathophysiology. The most significant PBM effects on mitochondrial morphology and network parameters were observed at 652 and 689 nm (15 mW/cm^2^ for 180 s). These conditions affected all the measured mitochondrial parameters considered, except the aspect ratio, indicating changes in mitochondrial size, shape, and connectivity. While 808 nm also had an impact, the effects were less pronounced. The 730 nm wavelength had no discernible effect on mitochondrial morphology. The lack of a significant PBM effect on the aspect ratio at all conditions can be attributed to its specific dependence on elongation. At the same time, the form factor is more sensitive to changes in the mitochondrial shape [48]. Mitochondrial dysfunction can significantly impair cellular energy production by disrupting the electron transport chain and reducing ATP availability, ultimately hindering processes critical for angiogenesis, cell migration, and wound healing [49,50]. PBM has demonstrated the ability to enhance mitochondrial function, often by increasing OCR and supporting mitochondrial biogenesis, thereby boosting cellular energy reserves [4,13]. In this study, using a mitochondrial stress test in the HUVECs, the 689 nm wavelength surprisingly outperformed 652 nm in stimulating OCR, an effect that also extended to mitochondrial morphology and network dynamics (Figure 5). This result was particularly unexpected, as previous analyses consistently showed 652 nm to be more effective across all the studied parameters. The increased OCR and enhanced mitochondrial structural dynamics observed with 689 nm may point to wavelength-specific interactions within mitochondrial complexes, especially those associated with CcO [12]. Despite 652 nm’s superior performance in stimulating cell activity across multiple markers, the observed advantage of 689 nm in OCR suggests potential for differential wavelength effects based on specific cellular functions and mitochondrial adaptations [12,51]. This outcome emphasizes that the choice of wavelength can have nuanced effects, where even similar wavelengths might preferentially support different cellular processes under PBM.

It should also be noted that due to potential light scattering and refraction within the plate, a minimal amount of “crosstalk light” (<6%) may have reached control/sham wells. To address this possible issue, control experiments performed with physically separated Petri dishes confirmed that this level of light exposure generated no measurable effects [18].

Overall, our findings highlight the positive PBM effects on cell migration, proliferation, and mitochondrial metabolic activity when optimal illumination conditions are applied. Our results also demonstrate that PBM performed at 730 nm in our conditions does not produce measurable effects, aligning with previous studies [4,8,9,17,18]. These results, combined with our observed responses, underscore the importance of precise light dosimetry for successful PBM treatments, particularly when treating “bulk” (larger than several mm^3^) tissues in which the light distribution is heterogeneous.

## 4. Materials and Methods

### 4.1. Cell Culture

Pooled human umbilical vein endothelial cells (HUVECs), obtained from Lonza (Basel, Switzerland), were cultured in Endothelial Growth Medium-2 BulletKit (EGM-2, Lonza, Switzerland) on gelatin-coated plates (1% gelatin type B, Sigma, Switzerland) as previously detailed [52]. The cells were maintained in a humidified incubator at 37 °C with 5% CO_2_ (Sanyo CO_2_ incubator, Labtec services Wohlen AG, Switzerland).

Human vascular smooth muscle cells (VSMCs) were isolated from human great saphenous vein segments as previously reported [53]. Vein explants were cultured on gelatin-coated plates (1% gelatin type B, Sigma, Buchs, Switzerland) in RPMI 1640 medium supplemented with 10% fetal bovine serum (FBS, Sigma, Switzerland), and 1% antibiotic-antimycotic solution (penicillin G 10,000 U/mL, streptomycin sulfate 10,000 U/mL). The cells were incubated at 37 °C with 5% CO_2_.

For both cell types, experiments were performed using passages 3–8.

### 4.2. PBM Illumination Protocol In Vitro

Continuous wave (CW) light sources at 652, 689 (CeramOptec, Bonn, Germany), 730, and 808 nm (Roithner LaserTechnik GmbH, Vienna, Austria) were employed. The illumination time was fixed at 180 s while the irradiances varied from 1.5 to 25 mW/cm^2^. The wavelengths and illumination durations were selected based on reports from other groups and our own data [4,17,38,54]. The samples (12-well plates or Petri dishes) were evenly illuminated using a frontal light distributor (Lausanne, Medlight SA, Model FD1, Switzerland), placed 15.5 cm from the samples, and coupled to the light source, producing a circular, uniform light spot (diameter: 9.8 cm). All the experiments included a control group (no PBM) and a PBM group exposed to specific light conditions.

### 4.3. Wound Healing Assay

The HUVECs and VSMCs were seeded to confluence in 12-well plates (Corning, Lucerne, Switzerland). A scratch wound was created using a sterile 200 µL pipette tip, followed by media replacement. The cells were then illuminated, and initial phase contrast images were captured (t = 0 h). The plates were returned to the incubator, and subsequent images were acquired after 8 h (HUVECs) or 24 h (VSMCs) to assess wound closure. The observation times after creating the wound with a pipette tip were determined through the empirical assessments of cell migration for each cell line. The ImageJ 1.52a software (NIH, Bethesda, MD, USA) was used to calculate the relative wound closure surface, expressed as the ratio PBM/no-PBM.

### 4.4. BrdU Assay

The HUVECs were seeded in 35 mm imaging dishes with polymer coverslips (Ibidi, Gräfelfing, Germany) and incubated overnight with 10 µM BrdU before the PBM treatment. The cells were fixed in ice-cold methanol for 10 min and immunostained for BrdU as previously described [53,55,56]. Images were captured using a Nikon Eclipse 90i microscope. Total DAPI- and BrdU-positive nuclei were automatically quantified with the ImageJ 1.54f software [55].

### 4.5. ALA and Rhod 123 Administration and Fluorescence Imaging

To assess endogenous PpIX production, 24 h after the PBM illuminations, the cells were incubated with 1 mM 5-aminolevulinic acid (ALA, Sigma, Buchs, Switzerland) for 3 h. Given the pH of both EGM and RPMI (7.2–7.4), pH adjustment was unnecessary. Then, ALA was removed and the cells were washed twice with phosphate-buffered saline (PBS, ThermoFisher Scientific, Reinach, Switzerland) before replacing the medium with ALA-free media for PpIX fluorescence intensity measurements.

The mitochondrial potential was monitored using 2.6 µM Rhod 123 (Sigma, Switzerland) for 30 min at 37 °C. The cells were then washed twice with PBS before replacing the medium with EGM or RPMI for immediate visualization.

An epifluorescence microscope (Nikon, Labophot-2, Tokyo, Japan) equipped with a 10× objective (NA: 0.30, WD 16.0 mm, Nikon, Japan) was used for both PpIX and Rhod 123 imaging. A BV-2A Nikon filter cube (455 nm dichroic mirror, excitation: 400–440 nm, emission: 470 nm high pass filter) combined with an E610LP filter (Chroma Technology, Vermont, N. Eng., Bellows Falls, VT, USA) was used to observe the PpIX fluorescence. A FITC Nikon cube (505 nm dichroic mirror, excitation: 480/30, emission: 535/45) was used to observe the Rhod 123 fluorescence.

### 4.6. Semi-Automatic Cell Segmentation Method of Microscopic Images for Quantification of PpIX

**Figure 7:**
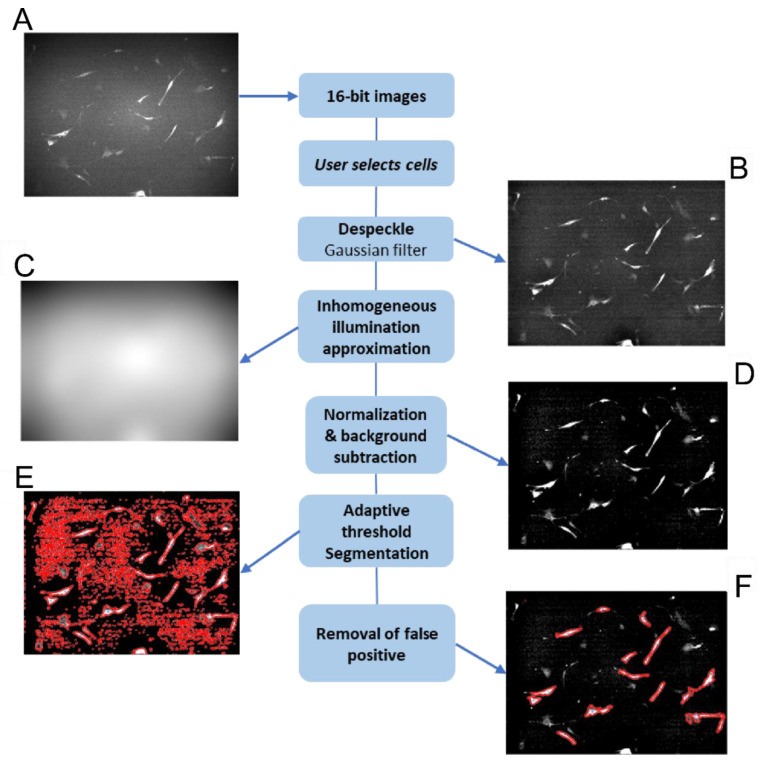
Image processing workflow. (**A**): Original 16-bit image. (**B**): Noise-reduced image after despeckling. (**C**): Extracted background illumination pattern. (**D**): Background-corrected image. (**E**): Detected regions of interest (ROIs). (**F**): Final image with non-selected ROIs removed.

A semi-automatic pipeline was developed for PpIX and Rhod 123 fluorescence analysis, employing Bradley’s adaptive thresholding for cell segmentation and quantification. This MATLAB-based pipeline with its GUI development environment, visualized in Figure 7, required initial manual cell selection within image frames to ensure complete cell analysis (approximately 20 cells per image). Background illumination correction was achieved by sampling cell-free areas. The high-frequency noise was removed using a Gaussian filter with a small kernel. The inhomogeneous background illumination was approximated by using a Gaussian filter with a large kernel and used to normalize cell intensities across the image (Figure 7B,C). Cell segmentation was performed using mean adaptive thresholding (Bradley’s method [57]), with adjustable sensitivity and neighborhood size parameters. Only user-selected cells were analyzed post-segmentation. Besides qualitative assessment through visual inspection, a tool aided users in selecting the optimal adaptive thresholding parameters by evaluating the detection success rate of the selected cells across various parameter ranges.

### 4.7. Rhod 123 Fluorescence Lifetime Imaging in HUVECs

The HUVECs were seeded in 35 mm Petri dishes (Corning, Switzerland) and exposed to PBM (15 mW/cm^2^, 180 s) at 652, 689, 730, or 808 nm. After 24 h, the cells were stained with 2.6 µM Rhod 123 for 30 min (Section 4.5) before fluorescence lifetime imaging microscopy (FLIM). FLIM was performed, as previously reported [18], using a MicroTime 100 confocal microscope (PicoQuant, Berlin, Germany) equipped with a 440 nm pulsed laser. A 40× objective (LUMPLFLN40XW, Olympus, Tokyo, Japan) captured images with an 80 µm × 80 µm field of view. Fluorescence emission (545 ± 40 nm) was detected by a single-photon counting detector. Pixel decay times were fitted to a mono-exponential model using the SymPhoTime 64 software (PicoQuant, Berlin, Germany). For each PBM condition, FLIM data from 18 regions of interest (ROIs; 3 per cell, 6 cells) were analyzed. Each lifetime image represented a single HUVEC cell, with three ROIs chosen per cell. Mean Rhod 123 fluorescence lifetimes were calculated across all the selected ROIs and cells. For each PBM condition, at least six FLIM images, totaling a minimum of 18 ROIs, were analyzed.

### 4.8. Mitochondrial Morphology Analysis

To assess the effects of PBM on mitochondrial state and function, we utilized a comprehensive pipeline integrated with an ImageJ plugin [48]. A multiplexed analysis was conducted using intensity images acquired with the MicroTime 100 upright time-correlated single photon counting (TCSPC) confocal fluorescence microscope mentioned above. This investigation aimed to examine the impact of specific PBM illumination conditions on mitochondria and their network in HUVECs. The visual inspection of mitochondrial structures and adaptive thresholding of the images using an open-source image plugin integrated with the ImageJ platform (NIH, Bethesda, USA) [48] were employed for this analysis.

### 4.9. Seahorse

Mitochondrial stress tests were assessed in confluent HUVECs using the Seahorse XF Cell Mito Stress Test Kit (Agilent Technologies, Inc., Seahorse Bioscience, Basel, Switzerland) according to the manufacturer’s protocol. The Seahorse experiments were conducted in a 96-well plate, with the conditions (no PBM, 652 nm, 689 nm, and 730 nm; 15 mW/cm^2^ during 180 s) separated by black screens dividing the well plate into 4 groups. Data were analyzed using the Seahorse Wave Desktop 2.6.1 Software.

### 4.10. Statistical Analysis

Data are presented as the mean ± standard error of the mean (SEM). The statistical analysis was performed using OriginPro 2018. The normal distribution of data was assessed using the Shapiro–Wilk test and Kolmogorov–Smirnov test. All data had a normal distribution. One-way ANOVA was used for intergroup comparisons, followed by Tukey’s post hoc test for multiple comparisons at a significance level of *p* < 0.05.

### 4.11. Ethics Statement

Human great saphenous veins were obtained with written informed consent from donors undergoing lower limb bypass surgery [58] following the protocols approved by Lausanne’s University Hospital and the Cantonal Human Research Ethics Committee (http://www.cer-vd.ch/ (accessed on 25 December 2024), Protocol Number 170/02). These protocols adhere to the Declaration of Helsinki (1975, revised 1983) regarding human tissue use.

## 5. Conclusions

This study thoroughly assessed the effects of various PBM illumination conditions on multiple cellular parameters reflecting the metabolic activities of HUVECs and VSMCs, identifying the most effective wavelengths (652 and 689 nm) and irradiances (15 mW/cm^2^) for enhancing cell migration, proliferation, endogenous PpIX production, mitochondrial function, and morphology. Our results highlight PBM’s significant therapeutic potential for wound healing and related conditions. This study also stresses that optimizing and mastering the radiometric parameters is essential to fully harness PBM’s benefits. By precisely controlling factors such as the irradiance, the fluence rate, which changes with depth, and the light dose, PBM will probably be successfully implemented across a range of clinical applications, presenting promising opportunities for promoting healing and improving patient outcomes.

## Data Availability

Data is contained within the article.
